# Beirut Ammonium Nitrate Explosion: A Man-Made Disaster in Times of the COVID-19 Pandemic

**DOI:** 10.1017/dmp.2020.451

**Published:** 2020-11-18

**Authors:** Mazen J. El Sayed

**Affiliations:** 1 Department of Emergency Medicine, American University of Beirut Medical Center, Beirut, Lebanon; 2 Emergency Medical Services and Prehospital Care Program, American University of Beirut Medical Center, Beirut, Lebanon

**Keywords:** ammonium nitrate, Beirut, COVID-19 pandemic, disaster response, explosion

## Abstract

The largest non-nuclear blast in modern history took place on August 4, 2020, at 6:07 PM in Beirut, Lebanon, after an estimated 2750 tons of unsafely stored ammonium nitrate exploded. The physical and social impacts of this catastrophic event coinciding with the coronavirus disease (COVID-19) pandemic were massive. This article describes the national and international emergency responses to this event and highlights the impact of the explosion on the health care sector in Lebanon. Challenges noted during this response with recommendations for improving response to future disasters are also described.

## Event

The largest non-nuclear explosion in modern history took place on August 4, 2020, at 6:07 PM in Beirut, Lebanon. This catastrophic event started 30 minutes earlier with a fire in a large hangar where fireworks and an estimated 2750 tons of ammonium nitrate were unsafely stored in an urban setting in the capital of Lebanon.^1^ Several small explosions were followed by a massive explosion that resulted in over 6000 injured, 200 fatalities, and over 100 missing casualties. It also destroyed a major part of the city with an estimate of over US $10 billion in infrastructure damage leading to over 300 000 displaced individuals. At the time of this report, the direct cause of this incident was still being investigated and theories ranged from a deliberate attack on the storage site to an accidental explosion of a large volume of explosive chemical material stored in an unsafe manner. The chemical cargo was present in the hangar for over 6 years after the Lebanese Port authorities confiscated it from a ship that entered the port to transport additional cargo but, for presumed technical reasons, the chemical cargo was unable to proceed with its journey from Georgia to Mozambique.

## Initial Response

The fire in the hangar prompted the Beirut Fire Department to respond to the incident site with first responders unaware of the presence of explosive material in the Hangar. The fire, which was filmed by many bystanders, was followed by a large explosion and a blast wave that was felt across Beirut. The explosion was heard over 200 km in the island of Cyprus and registered seismic waves equivalent to a magnitude of a 3.3 earthquake.^2^

Prior to the blast, there were no notifications to public or to other agencies to allow for population protection in terms of rapid evacuation or sheltering in place.^3^ This event immediately overwhelmed the ability of Beirut hospitals, emergency medical services (EMS) agencies, first responder agencies, and other responding agencies to mount an effective response. Casualties flooded local hospitals with the less injured arriving first by private transportation or by walking to nearby hospitals and the more injured arriving later by EMS vehicles. EMS agencies headquarters and vehicles in Beirut were also affected by the blast. Initial communication between hospitals and between hospitals and EMS was lacking since all hospitals within a 5-mile radius from the explosion site were affected at the same time. Patients presented mainly with blast injuries seen with high order explosives. These included traumatic brain injuries, penetrating and blunt injuries from glass and flying debris, globe (eye) injuries, and musculoskeletal injuries (including fractures and contusions). Very few patients presented with burns. Patients with minimal injuries visited several hospitals before being attended to.

## Scaling Up of Response

The Government of Lebanon, realizing the massive impact of this incident, declared a state of emergency and launched an immediate appeal for disaster assistance to the international community. At the same time, the Lebanese Army formed an incident command structure that would oversee the disaster response activities, including operations, logistics, planning, and finance and administration. This response model was previously described in an article evaluating national emergency response to mass casualty incidents in Lebanon^4^ and recommending the adoption of principles of the US National Incident Management System.^5^ Drills were also conducted in previous years in collaboration with non-governmental organizations (NGOs) and the United Nations Interim Force in Lebanon.

## Initial Disaster Operations

The Lebanese Army secured the blast area perimeter to allow for investigation teams and for search and rescue operations to proceed immediately after the blast. The Beirut Municipality was tasked in coordination with the High Relief Commission to assess damages in affected areas of Beirut. The Internal Security Forces were designated as the main agency to help with searching for missing individuals and to conduct forensic/DNA identifications of unidentified victims/body parts in coordination with the Lebanese Red Cross. They were also charged with maintaining security and safety in affected areas by preventing looting and theft from partly destroyed houses and businesses.

A liaison committee was also formed between the Lebanese Army and the Ministry of Health to prioritize medical needs and to oversee donations and coordinate the timely delivery of resources.

The incident command center also asked media outlets to rely only on official communications released by designated media liaison officers. A dedicated call center was launched for all public inquiries related to the response activities. Another task force was assigned to work on disaster recovery activities, including resuming operations in the unaffected part of the port to allow for receiving international assistance. Beirut International Airport was also designated as the main site for receiving international assistance teams and resources.

## Response Priorities

### Search and Rescue Operations

Search and rescue operations for missing individuals at the blast site were the initial response priority. The initial operations were conducted by the Lebanese Civil Defense (main government agency tasked with urban search and rescue), the Lebanese Army, and other responding agencies. Several international teams arrived to Lebanon the day after the explosion and were assigned specific sectors and initiated work on-site with advanced equipment and rescue dogs. The blast site map identified at least 7 international teams from Russia, France, Poland, Qatar, Netherlands, Greece, and Czech Republic. Other teams, including the Turkish team, arrived a few days later and started working on the expanded blast site. An initial estimate of over 100 individuals missing was reported after the blast.

### Health Care Sector Impact Assessment

A quick assessment of the impact of the event on the health care system, addressing the needs of the injured, and regaining the pre-incident capacity of the system were the main medical priorities. The direct physical impact on the health care system consisted of damages to hospitals and to other health care entities: Three major hospitals (St. George Hospital University Medical Center, Wardieh Hospital, and Karantina Hospital) became completely nonfunctional resulting in the total loss of approximately 500 beds. Three other hospitals sustained partial damage (Geitawi Hospital, LAU Medical Centre–Rizk Hospital, and American University of Beirut Medical Center).^6^ The largest national stock of medications was also partly affected and 17 containers of medical supplies located at the port were partly destroyed. Several medical providers were also among the blast victims with several deaths reported from hospitals that were close to the blast site. According to the Lebanese Order of Physicians, over 2000 doctors’ offices and clinics were also affected by the explosion.

At the time of the blast, the health care sector in Lebanon was already experiencing tremendous challenges in terms of capacity and resources. A deepening economic and financial crisis that started in 2019 caused several health care organizations to reduce their operations and their workforce in order to cope with currency devaluation, price inflation, and capital control measures that were implemented.^7^ Most hospitals were also prioritizing preparedness for the coronavirus disease (COVID-19) pandemic with 4022 cases registered in Lebanon 1 day before the blast.^8^

Despite all of these challenges, local hospitals received over 6000 casualties and depleted most of their supplies to respond to this disaster. Hospitals that were severely hit during the blast had to relocate their patients to other facilities, which resulted in increased occupancy in most hospitals in Beirut and reduced their ability to cope with a concurrent surge in COVID-19 cases requiring hospitalization. Caring for patients during this response compromised precaution practices that were implemented in most hospitals related to the COVID-19 pandemic, such as removing COVID-19 screening and testing requirements and reduced personal protective equipment (PPE) standards during advanced airway management.

Field hospitals were part of the international assistance and mutual aid delivered by other countries.^9^ By August 12, 2020, there were at least 5 international hospitals operating in the greater Beirut area: Most were set up to operate independently and were run by military health care teams. These included the Russian, Jordanian, Moroccan, and Iranian hospitals. The Qatari hospital was set up to support the functions of St. George Hospital, which was located near the blast site. Most had general capabilities for treating injured victims and providing minor surgeries with few intensive care beds available in the Iranian and Qatari hospitals. Two hospitals also had laboratory COVID-19 polymerase chain reaction (PCR) testing (Russian and Jordanian) and pharmacies for chronic medications (Iranian, Jordanian, and Qatari). Treatment of mental health emergencies was also offered at the Russian hospital. None of the hospitals were designed to care for or admit patients with COVID-19.

Donations received by the Lebanese Government included large stocks of medical supplies and medications and field hospitals. International health care organizations, including the World Health Organizations, US Agency for International Development, and United Nations High Commissioner for Refugees, also dispatched emergency response teams to Lebanon to help assess the situation and address needs of both private and public entities in the health care sector.

Other NGOs and the private sector focused directly on the needs of the displaced and affected individuals. There were several donation funds established for different causes on social media outlets to support different initiatives, including treating children, rebuilding houses, and funding shelters. Support of government agencies by the private sector was non-existent for several reasons: (1) lack of trust between the Lebanese people and the government because of the financial crisis that was mainly attributed to corruption, and (2) blaming of the government for negligence in addressing the unsafe storage of the ammonium nitrate.

#### Challenges During This Response

Several challenges were observed during the response to this catastrophic event that coincided with the COVID-19 pandemic.

At the mitigation level, government agencies failed to adequately identify the hazard risk associated with the storage of large volumes of explosive chemicals in an urban site. Ammonium nitrate is a fertilizer but is also used as a blasting agent and is classified as an explosive when it mixed with more than 0.2% of combustible substances. It is also known to have caused some of the most destructive accidental explosions in the past.^10^ Despite the presence of command and control regulatory requirements for importation, storage, and transportation of chemical products in Lebanon, awareness and decisive actions to reduce the hazard risk, in this case, were missing. As a result, the Beirut Fire Department, the primary responding agency in Beirut, responded to a fire without prior knowledge of the stored material. Ten firefighters lost their lives in the blast. Additionally, an expedient emergency assessment at the start of the fire was also not adequate since no notifications were sent to the public to protect the population in the vulnerable zone. Bystanders were filming the fire from their houses in close proximity to the blast site for 30 minutes prior to the explosion that destroyed their homes and left many of them injured or dead. Similarly, hospitals, emergency response agencies, and other operating businesses did not receive any notification about a potential catastrophe. A disaster management authority in charge of risk assessment, hazard identification, and disaster response is missing in Lebanon because the disaster response draft law that was submitted in 2012 to the Lebanese Parliament has not been approved to date.^11^

At the response level, previous drills helped with the immediate establishment of the incident command center and the Lebanese army assuming command of the overall response. The immediate call for assistance launched by the government helped scale up quickly the response with much needed search and rescue teams with adequate skills and equipment arriving within hours to Lebanon and launching operations as of the next morning of the explosion. The incident command structure allowed for responding agencies to integrate into a response framework with clearly delineated roles and responsibilities. A major challenge the government faced in this response was related to the role of the private sector and the community response with several NGOs asking the international community to bypass the government agencies because of concerns for corruption and the deep political crisis that Lebanon was facing at the time of the event. In fact, the international coalition for helping Lebanon raised over US $200 million and requested that funds go directly to the Lebanese population while mandating reform measures from the Lebanese Government.^12^

Challenging aspects of the medical response were many: (1) The blast affected most neighboring hospitals in Beirut, which resulted in lack of information about the functional status of hospitals and lack of initial coordination between hospitals or between EMS agencies and hospitals. Several hospitals faced difficulties with coping with the initial surge of patients immediately after the explosion and referred many patients with minor injuries to more distant hospitals; (2) fatality management was also problematic with morgues at local hospitals filled immediately by casualties. Requests were sent from hospitals to EMS agencies to define a protocol for fatality management during the response and to identify alternative locations for storage of dead bodies; (3) unconscious patients’ identification was another challenge with many patients arriving with severe traumatic brain injuries or with life-threatening injuries and getting intubated or going straight to the operating rooms without the ability to identify them. Delays with fatalities’ identification were also present, and by August 10, 2020, 20 out of a total of 152 fatalities were still unknown. Potential contributing factors related to this delay were the massive scale of the event and the presence of a large number of refugees in Lebanon.

Another challenge in the overall medical response was related to the role of the field hospitals. The existing Lebanese hospitals were able to absorb quickly the surge of patients and to address over subsequent days most of the initial needs of injured patients. Patients also followed up at the hospitals where they were initially treated for their return visits. Independently deployed hospitals started receiving general medical visits, and only 1 hospital was paired with another existing partly destroyed hospital to support its functions. The functions and capabilities of these hospitals were not initially coordinated with existing hospitals, and their locations and services were not immediately clarified to the public. Several disaster medical assistance teams reached Lebanon and, after a quick assessment, realized that the needs were mainly in stocks, supplies, and medications rather than medical personnel or sites of care.

Coordination and deployment of resources were initially slightly delayed, especially that the call for international assistance did not identify a list of urgent medical needs. The response priorities were later clarified by the incident command center to allow for international assistance teams to deploy resources in a more effective manner. The Ministry of Health dispersed gradually medical supplies to local hospitals.^13^ Donations also reached different entities in Lebanon from multiple international sources, despite the existing structure of the incident command center to centralize this and avoid duplication of resources.

Public information and communication remained major challenges during this disaster response. Issues ranged from missing pre-explosion notifications to the absence of official status reports on response activities, such as daily updates on rescue operations, fatalities’ identification, damage assessment, international assistance, and on other disaster recovery-related activities. Multiple NGOs worked on filling this gap by launching public databases for missing individuals, unidentified victims, lists of available resources (food and shelter), and so forth.

Table 1 lists recommendations for improving response, recovery, and resilience to future disasters in Lebanon.

**Table 1.** Recommendations for improving response, recovery, and resilience for future disasters

**Prevention and Protection**	Establish a system for intelligence- and information-sharing between agencies and hospitals.
	Formalize a mechanism for public information and warning in routine events and disasters.
	Search for and detect storage sites of hazardous materials.
	Identify vulnerable areas in the community and enforce protective measures against disasters.
	Define critical infrastructure in the health care sector and introduce protective measures against blasts and other types of disasters.
**Mitigation**	Develop and implement a hazard-specific mitigation plan at the national level
	Test communication with public through early notifications for evacuation and or sheltering.
	Improve information-sharing system with responding agencies and hospitals
	Continue multi-agency drills for disaster response and involve stakeholders from private sectors and communities.
	Hazard risk assessment to identify storage sites of hazardous material and enforcing regulations.
	Risk stratification of zones for future events.
	Develop a plan and practice tools for a rapid assessment post disaster.
	Pre-define life-saving and life-sustaining activities at the community level.
**Response**	Continue to improve the different branches of the incident command structure.
	Identify immediate response priorities and gaps and communicate these clearly to local and international response agencies.
	Establish a critical information network and improve efficiency of information-sharing across different levels of government and to responding agencies.
	Introduce disaster notifications to the public, hospitals, and to other stakeholders.
	Initiate and organize government and community search and rescue operations.
	Quick assessment of the scale of the event and determine need for scaling up the response.
	Quick assessment of the status of critical infrastructure and prioritize re-establishing this infrastructure.
	Assess status of critical transportation channels, continuing threats, and ensure access to disaster zone for responding agencies.
	Clarify mechanism for integrating international agencies into overall response framework (responding agencies and field hospitals).
	Ensure ability to deliver acute care for a large number of victims, and prioritize activities to increase surge capabilities.
	Clearly define roles and responsibilities and involve public sector and NGOs in a formal way.
	Mobilize resources after a quick assessment to acute care facilities and to disaster victims.
	Ensure ability to continue to deliver life-saving and life-sustaining activities at all levels.
	Establish a system for fatality management.
	Establish mechanism for identifying unconscious victims who are hospitalized.
	Establish an effective system for mobilizing resources to responding agencies, facilities, and to disaster victims.
	Establish information centers for the public and for disaster victims.
	Define owners of different response activities, including establishing emergency shelters, reporting of, and locating missing individuals.
	Ensure protection to site and safety (person, property, and environment, including assessment for residual threats related to CBRNE* incidents).
	Clarify a transparent mechanism for receiving aid and donations and for distributing donations.
	Create a formal vetting mechanism for old and new NGOs involved in the response.
**Recovery**	Define a recovery plan that covers all sectors.
	Establish integrated leadership for all recovery activities with clear goals and time lines.
	Prioritize recovery of the health care sector and other health/social services.
	Improve communication and information dissemination about the recovery status of affected services.
	Clarify access to information-related and all types of recovery activities.
	Create a feedback mechanism about the overall status of recovery operations for involved stakeholders.
	Define mechanisms for ensuring sustainability in recovery activities in terms of balancing needs and availability of resources.

*CBRNE = chemical, biological, radiological, nuclear, and high yield explosives.

This catastrophic explosion occurred during the COVID-19 pandemic. The number of COVID-19 cases was starting to rise quickly in July after the reopening of different sectors, including the Beirut International Airport. The health care system capacity, however, was being challenged by the financial crisis, the reduction in workforce, and closure of COVID-19-dedicated wards and intensive care units (ICUs) in private hospitals because of difficulties in securing resources, such as PPE, ventilators, and dialysis equipment. The main challenge of the health care system after the explosion was the reduction in capacity to cope with the surge of COVID-19 cases, especially that 2 large, affected hospitals were also treating COVID-19 patients. In fact, the number of COVID-19 cases rose to reach 10 347 total cases by August 19, 2020, with post-explosion cases accounting for around 61% of total cases. Several factors may explain this surge: (1) Injured patients were treated at different hospitals, and precaution standards were compromised during the response; (2) mass gatherings were frequent to help with urban search and rescue operations and riots started immediately after the explosion, holding the government accountable for negligence; and (3) The natural progression of the pandemic since the weekly COVID-19 reproduction rate average exceeded 1.5 in the pre-explosion phase. This surge in COVID-19 cases required that the priorities of the response be shifted back to the COVID-19 response with the decision to reimplement a partial lockdown while allowing recovery operations to continue. Requests for help and for resources are also prioritizing the COVID-19 response in terms of building ICU capabilities in different Lebanese hospitals and modifying field hospitals to be able to accommodate COVID-19 patients. An official assessment of the status of the health care sector in Lebanon post-Beirut explosion is still pending.

In summary, the emergency response to the ammonium nitrate explosion in Beirut on August 4, 2020, was the first reported international response to a man-made disaster during the COVID-19 pandemic. Its physical impacts (casualties and damage) and social impacts (psychological, economic, and political) are massive. The government responded with a clear incident command structure, and gaps were filled by the private sector and the community. Man-made disasters are unfortunately very frequent in Lebanon, and the time for preparedness and mitigation seems to not be enough. Lebanon needs all the help from the international community to be able to recover fast from this disaster and to mount an effective response against the most imminent threat, which is the COVID-19 pandemic.
